# The prediction of 24-h mortality by the respiratory rate and oxygenation index compared with National Early Warning Score in emergency department patients: an observational study

**DOI:** 10.1097/MEJ.0000000000000989

**Published:** 2022-12-05

**Authors:** Bart G.J. Candel, Bas de Groot, Søren Kabell Nissen, Wendy A.M.H. Thijssen, Heleen Lameijer, John Kellett

**Affiliations:** a Emergency Department, Maxima Medical Centre, Veldhoven, Noord-Brabant; b Emergency Department, Leiden University Medical Centre, Leiden, Zuid-Holland, the Netherlands; c Institute of Regional Health Research, Center South-West Jutland, University of Southern Denmark, Esbjerg; d Department of Emergency Medicine, Odense University Hospital, Odense, Denmark; e Emergency Department, Catharina Hospital, Eindhoven, Noord-Brabant; f Department of Emergency Medicine, Medical Centre Leeuwarden, Leeuwarden, the Netherlands

**Keywords:** clinical prediction rule, early mortality, mortality prediction, respiratory rate oxygen saturation index

## Abstract

The ROX index combines respiratory rate and oxygenation to predict the response to oxygen therapy in pneumonia. It is calculated by dividing the patient’s oxygen saturation, by the inspired oxygen concentration, and then by the respiratory rate (e.g. 95%/0.21/16 = 28). Since this index includes the most essential physiological variables to detect deterioration, it may be a helpful risk tool in the emergency department (ED). Although small studies suggest it can predict early mortality, no large study has compared it with the National Early Warning Score (NEWS), the most widely validated risk score for death within 24 h.

**Aim:**

The aim of this study was to compare the ability of the ROX index with the NEWS to predict mortality within 24 h of arrival at the hospital.

**Methods:**

This was a retrospective observational multicentre analysis of data in the Netherlands Emergency Department Evaluation Database (NEED) on 270 665 patients attending four participating Dutch EDs. The ROX index and NEWS were determined on ED arrival and prior to ED treatment.

**Results:**

The risk of death within 24 h increased with falling ROX and rising NEWS values. The area under the receiving operating characteristic curves for 24-h mortality of NEWS was significantly higher than for the ROX index [0.92; 95% confidence interval (CI), 0.91–0.92 versus 0.87; 95% CI, 0.86–0.88; *P* < 0.01]. However, the observed and predicted mortality by the ROX index was identical to mortality of 5%, after which mortality was underestimated. In contrast, up to a predicted 24-h mortality of 3% NEWS slightly underestimates mortality, and above this level over-estimates it. The standardized net benefit of ROX is slightly higher than NEWS up to a predicted 24-h mortality of 3%.

**Conclusion:**

The prediction of 24-h mortality by the ROX index is more accurate than NEWS for most patients likely to be encountered in the ED. ROX may be used as a first screening tool in the ED.

## Introduction

In 2016, Roca *et al*. proposed an index combining respiratory rate and oxygenation (ROX) to predict the success of oxygen therapy by high-flow nasal cannula in pneumonia patients with acute hypoxemic respiratory failure [1]. The ROX index is calculated by dividing the patient’s oxygen saturation, by the inspired oxygen concentration (FiO_2_), and then by the respiratory rate. Only a handful of studies have evaluated the ability of the index to predict adverse outcomes in acute diseases, such as coronavirus disease 2019 (COVID-19) and sepsis [2–4]. However, one small preliminary study from a Canadian teaching hospital and a low-resource hospital in Africa looking at three markedly different unselected patient populations found the ROX index to be a powerful discriminator of early mortality [5].

The National Early Warning Score (NEWS) predicts deterioration from sepsis better than systemic inflammatory response syndrome or quick Sequential Organ Failure Assessment (qSOFA) score [6] and is the most widely validated risk score for death within 24 h [7,8]. However, the ROX index has been reported to predict the deterioration of COVID-19 patients earlier than NEWS when measured by composite outcomes [4]. The aim of this study was to compare the ability of the ROX index with NEWS to predict mortality within 24 h of arrival to the hospital of a large population of patients attending several Dutch EDs.

## Methods

### Study design and setting

This retrospective observational multicentre study analyzed data in the Netherlands Emergency Department Evaluation Database (NEED), a registry containing clinical data from all ED visits from the participating hospitals used to benchmark the quality of their care (www.stichting-need.nl). Only the vital signs and mental status measured at the beginning of ED presentation, before ED treatment, are recorded in the NEED, and only one set of vital signs is recorded per patient. Further details on the NEED have been previously published [9].

### Selection of participants

All consecutive ED patients ≥18 years attending four participating hospitals EDs were included. Inclusion periods varied from 1 January to 31 December 2019, 1 January 2019 to 12 January 2020, and for two hospitals from 1 January 2017 to 31 March 2022. Patients were excluded from the NEED if none or only one vital sign (SBP, heart rate, peripheral oxygen saturation, respiratory rate, or temperature) were registered. In EDs in the Netherlands and elsewhere, vital signs are not registered in all patients, and respiratory rate and temperature are the most frequently omitted. For example, vital signs are often not registered in patients with an ankle distortion or a single fracture, because they are at low risk of adverse events and are often discharged. Respiratory rate is often only registered if patients are considered critically ill by the nurse. In the excluded patients, vital signs were considered missing not at random which prevented the possibility of imputation.

### Data collection, default assumptions, and outcomes

Details of how data were collected have been previously described [9]. Briefly, demographic characteristics, type of arrival to the ED, triage category, vital signs, and laboratory tests, as well as disposition and mortality were collected. The primary outcome was death within 24 h of ED arrival; for the primary analysis, it was assumed that no patients discharged from the ED died within 24 h.

### Statistical analyses

Continuous patient characteristics were described as mean (SD) or median (interquartile range). Categorical data were presented as numbers with proportions [*N*, (%)]. Prior to analyses, missing data were substituted by multiple imputations by the chained equation procedure, after which imputation was feasible based on missing value analyses. To enhance the multiple imputation procedure, we also used urea, leukocytes, and intensive care unit admission as predictors in the imputation procedure. When ≥4 vital signs were missing, observations were excluded. These were considered non-randomly missing and despite a comprehensive set of variables in the multiple imputation procedure, including outcomes, we did not consider imputation to be feasible.

We obtained 20 estimates of the missing vital signs for each patient with five iterations each. Regression coefficients and intercepts across imputed sets were averaged to incorporate the variance introduced by the imputation procedure [10–12]. For each imputation set, we calculated the NEWS and the ROX index. The ROX index, rounded to the nearest integer, was calculated by dividing the patient’s oxygen saturation, by the FiO_2_, and then by the respiratory rate (e.g. 95%/0.21/16 = 28).

Predictive performance was compared between ROX and NEWS using the area under the receiving operating characteristic curves (AUC) with 95% confidence intervals (CIs) and calibration plots. The DeLong test was used to compare AUCs. To evaluate clinical usefulness, Decision Curve Analyses (DCA) were produced, summarizing standardized Net Benefits at a range of thresholds [13]. DCA plots the net benefit of each ROX or NEWS value against the risk encountered at that value. The net benefit for each value of ROX or NEWS tested = (true positives/all) – (false-positives/all × exchange rate), at that value. The exchange rate is determined by the risk at the chosen value; if 10%, the exchange rate = 0.1/(1–0.1), implying that the harm of not being identified is nine times larger than unnecessary identification as high risk. The net benefit was standardized to the prevalence of 24 h-mortality at each value. DCA identifies threshold values of net benefit at different mortality risks that justify the measurement of NEWS or ROX in all patients [14].

Internal validation was performed by split sample analysis based on a pre-COVID period (till 1 February 2020) and COVID period (all patients after 1 February 2020). Additionally, because we assumed that discharged patients did not experience the outcome, a sensitivity analysis was performed in which a randomly selected 0.04% of the discharged patients were considered deceased within 24-h. This percentage was based on reports in the literature [15]. All analyses were performed in R statistical software [packages dplyr (v1.0.7;2021), rms (v6.2;2021), mice (v45;2011)].

### Ethical approval

The study was approved by the medical ethics committee of the Máxima Medical Centre (nr. 21.007). The study conforms to the principles outlined in the Declaration of Helsinki. The study conforms with the transparent reporting of a multivariable prediction model for individual prognosis or diagnosis TRIPOD statement [16].

## Results

**Fig. 1:**
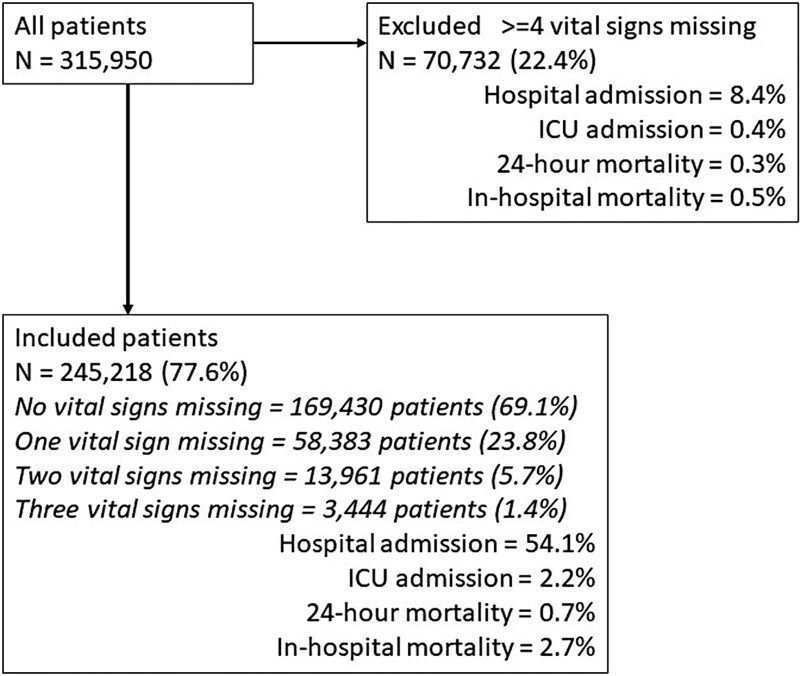
Included and excluded patients according to missing values, hospital and ICU admission, and 24 h and in-hospital mortality. ICU, intensive care unit.

During the study period out of 315 950 patients presented to the four participating EDs 70 732 (22.4%) were excluded because ≥4 vital sign values were missing. These excluded patients were younger, and significantly less likely to be admitted to the hospital or to die than the final population of 245 218 patients included in the study (Fig. 1). Most included patients (69.1%) had a complete set of the four classic vital signs and oxygen saturation, and only 7.1% had more than one missing value.

**Fig. 2:**
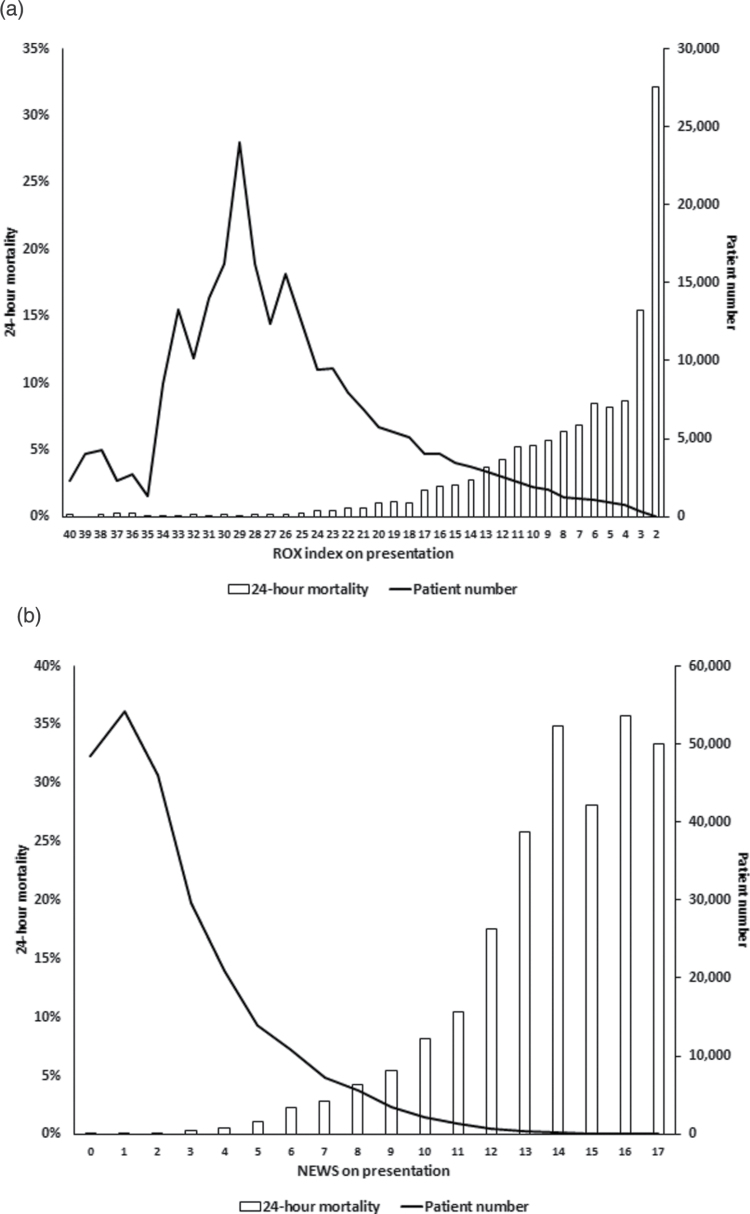
(a) Observed 24-h mortality and patient numbers according to ROX index determined at emergency department presentation. (b) Observed 24-h mortality and patient numbers according to NEWS values determined at emergency department presentation. NEWS, National Early Warning Score; ROX, respiratory rate and oxygenation.

The 1791 patients (0.7%) who died within 24 h were older, more likely to be men, and less likely to be alert (Table 1). High NEWS and low ROX index values were associated with 24-h mortality (Fig. 2a and b). The AUC for NEWS was significantly higher than for the ROX index (0.92; 95% CI, 0.91–0.92 versus 0.87; 95% CI, 0.86–0.88; *P* < 0.01).

**Table 1:** Age, sex, mental status, and vital signs of patients who died within 24 h, those who survived for 24 h and the total study population

	Total	Died	Alive	*P*	Imputed values
*N*	245 218	1791	241 944		75 788 (30.9%)
Male sex	123 966 (50.6%)	964 (53.8%)	122 208 (50.5%)	<0.0001	0 (0.0%)
Age (years)	61.3 SD 19.4	74.1 SD 13.8	61.2 SD 19.4	<0.0001	0 (0.0%)
	65.0 (49.0–73.0)	77.0 (68.0–84.0)	65.0 (48.0–76.0)		
SBP (mm Hg)	143.0 SD 21.7	133.0 SD 40.5	143.0 SD 26.5	<0.0001	3603 (1.5%)
	140.0 (125.0–159.0)	130.0 (102.0–159.0)	140.0 (125.0–159.0)		
Heart rate (beats per minute)	86.0 SD 21.2	95.5 SD 27.2	85.9 SD 21.1	<0.0001	8170 (3.3%)
	83.0 (71.0–97.0)	95.0 (76.0–113.0)	83.0 (71.0–97.0)		
Respiratory rate (breaths per minute)	17.9 SD 5.1	23.3 SD 9.4	17.8 SD 5.0	<0.0001	48 255 (19.7%)
	16.0 (14.0–20.0)	21.0 (17.0–28.0)	16.0 (14.0–20.0)		
Oxygen saturation (%)	96.7 SD 3.3	93.1 SD 7.8	96.7 SD 3.2	<0.0001	6249 (2.5%)
	97.0 (95.0–99.0)	95.0 (91.0–98.0)	97.0 (95.0–99.0)		
Temperature (°C)	36.9 SD 0.9	36.4 SD 1.5	36.9 SD 0.9	<0.0001	30 360 (12.4%)
	36.8 (36.3–37.3)	36.5 (35.8–37.2)	36.8 (36.3–37.3)		
Fraction of inspired oxygen (FiO_2_)	0.24 SD 0.10	0.38 SD 0.23	0.24 SD 0.10	<0.0001	121 561 (49.6%)
	0.21 (0.21–0.21)	0.29 (0.21–0.41)	0.21 (0.21–0.21)		
Alert^a^	74 926 (30.6%)	292 (16.3%)	74 202 (30.7.2%)	<0.0001	166 930 (68.1%)

Means, standard deviations (SD), medians and IQR are shown.

a Patients who needed to be aroused by voice or pain or were unresponsive were NOT alert – all other patients were considered ‘Alert’.

Approximately half of the study patients (133 019; 54.2%) presented before 2020 and the start of the COVID-19 pandemic. There was no difference between the AUC before and after the covid period for NEWS (0.92; 95% CI, 0.92–0.93 versus 0.91, 95% CI, 0.91–0.93; *P* = 0.12), and a small difference in AUC for the ROX index (0.86; 95% CI, 0.85–0.88 versus 0.88; 95% CI, 0.87–0.90; *P* = 0.02).

**Fig. 3:**
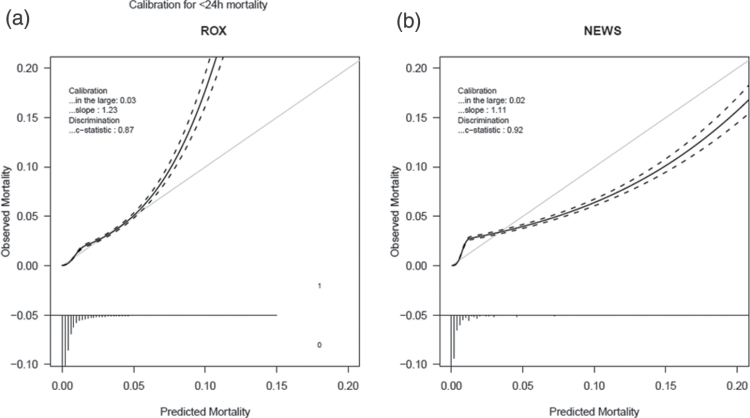
Calibration between predicted and observed 24-h mortality for ROX and NEWS. After discharge from the emergency department, 24-h mortality was assumed to be 0%. NEWS, National Early Warning Score.

**Fig. 4:**
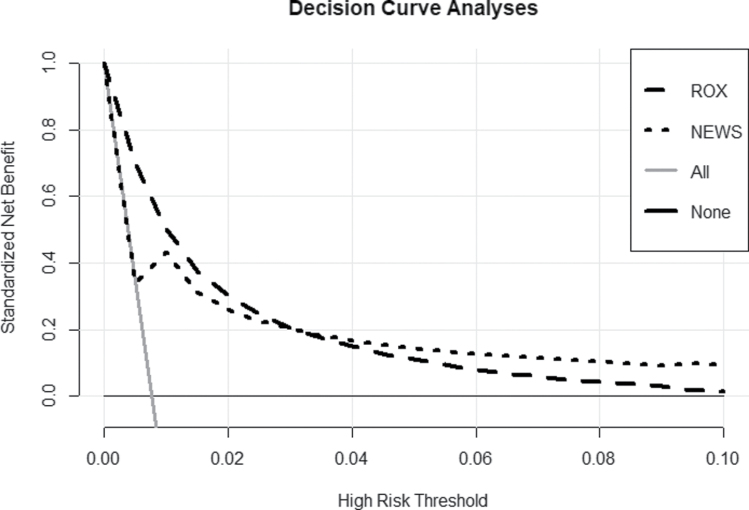
Decision Curve Analysis showing the standardized Net Benefit of ROX and NEWS at different predicted risks of 24-h mortality. After discharge from the emergency department, 24-h mortality was assumed to be 0%. NEWS, National Early Warning Score; ROX, respiratory rate and oxygenation.

Calibration of the ROX index was accurate up to an observed mortality of 5%, after which mortality was underestimated. In contrast, up to 24-h mortality of 3% mortality NEWS slightly under-estimates mortality, and above this level mortality was over-estimated (Fig. 3). Calibration for ROX improved at the start of the COVID-19 period compared to before, whereas NEWS remained unchanged (see Supplemental Data, Supplemental Digital Content 1, http://links.lww.com/EJEM/A354). The DCA graph shows that the ROX index has a higher net benefit than NEWS for predicted 24-h mortality below 3% (Fig. 4).

Sensitivity analysis showed that if the mortality within 24 h of discharged patients was 0.04% the AUC for ROX and NEWS were slightly reduced to 0.85 (95% CI, 0.84–0.86) and 0.89 (95% CI, 0.89–0.90), respectively. However, the predicted and observed 24-h mortality of ROX values now were the same up to a mortality rate of 6%, and the calibration of NEWS was unchanged (Supplemental Data, Supplemental Digital Content 1, http://links.lww.com/EJEM/A354); the ROX index continued to have a higher net benefit than NEWS for predicted 24-h mortality below 3%.

## Discussion

### Main findings

For 24 h mortality predicted to be below 3% (i.e. at ROX index ≥ 14), ROX has a higher net benefit than NEWS, while above a predicted 24 h mortality of 3% the net benefit of NEWS is higher. No difference was observed in the discriminative performance of either NEWS or the ROX index before and after the start of the COVID-19 pandemic. However, the calibration of the ROX index improved during the COVID-19 period.

### Strengths and limitations

Although it has the inherent limitation of retrospection, this is a large multicentre study of over a quarter of a million patients. Although a preliminary complete case analysis without imputation produced similar results to our final analysis, the judicious use of imputation reduced selection and other information bias [7]. For example, the FiO_2_ may not have been routinely recorded for many patients on room air, and excluding these patients would bias results toward patients who were more likely to be severely ill. As our primary outcome of interest was 24-h mortality, we did not exclude patients who were frequent ED attendees from the study. Unfortunately, ROX depends on respiratory rate measurements, which are often measured inaccurately [17–19] and we were not able to determine how the respiratory rate was measured in participating centers or verify the accuracy of these measurements. There was no follow-up of patients after ED discharge and our default assumption was that no discharged patients died within 24 h. However, sensitivity analysis showed that the results would not have been significantly changed even in the unlikely event of 0.04% of patients dying within 24 h of discharge [15,20,21].

### Interpretation

NEWS was specifically designed to identify patients at risk of imminent death and the risk of anyone dying within 24 h is extremely low [8]. Although our findings can only be applied with certainty to the population studied, there are very few reports in the literature of 24 h mortality above 1% in a general hospital and ED patients. In over 5 million observations reported in a systematic review of the literature, the overall mortality within 24 h was 0.6% and the AUC for 24-h mortality of NEWS was 0.897 [8]; only three studies [22–24] reported a 24-h mortality >1.0%, which ranged from 2.9% [23] to 10.2% [22]. Therefore, it is probable that our findings can be applied to most ED patients as very few are likely to have a 24-h mortality rate >3%. However, for severely ill and older patients who will inevitably require the measurement of a full set of vital signs NEWS may be more appropriate.

### Clinical implications

The higher observed discrimination of NEWS for 24-h mortality suggests that compared to the ROX index it is the superior test. However, the AUC is not useful for low-incidence outcomes such as 24-h mortality [25]. Additionally, discrimination does not indicate how close predicted mortality is to observed mortality. In this study, unlike NEWS, the 24-h mortality predicted by ROX was identical to the observed mortality up to a predicted mortality of 5.0%. Despite the lower AUC of ROX, DCA shows that the net benefit of ROX exceeds that of NEWS in patient populations with predicted 24-h mortality of less than 3%.

Respiratory rate may often not be measured or can be estimated, or even fabricated, especially if the patient is not thought to be ill [17]. This may hamper the implementation and adoption of ROX. However, devices are now available to measure respiratory rate continuously [26], and they can also be quickly and reliably obtained at the bedside using the free *RRate* smartphone application [27]. NEWS also requires measurement of respiratory rate and a full set of vital signs, which can take up more than 5 min to complete [28,29]. Therefore, ROX may be very useful in clinical practice as the initial method of risk assessment in ED patients. The index can be measured more rapidly and repeatedly at the bedside than NEWS and, therefore, could aid monitoring and decision-making in the acute setting for patients needing time-sensitive treatments, such as sepsis or trauma. However, if the predicted mortality is >3.0% (i.e. ROX falls below <14) additional vital signs should be measured to calculate NEWS, and the patient assessed further.

It remains to be seen if it is practical to adopt the ROX index as a routine method of ED risk assessment. Wider clinical use will require a cultural change, which recognizes the importance of the accurate measurement and recording of respiratory rate. Furthermore, early warning scores that are better calibrated over a wider risk range than either ROX or NEWS are needed to improve the risk assessment of all patients regardless of their clinical setting [30].

### Conclusion

In this patient population, the prediction of 24-h mortality by the ROX index is more accurate than NEWS for most patients likely to be encountered in the ED. ROX may be used as a first screening tool in the ED.

## Acknowledgements

The authors of this unfunded study would like to acknowledge the assistance and cooperation of Leiden University Medical Centre and the board of the Netherlands Emergency department Evaluation Database.

B.G.J.C., J.K., and S.K.N. devised and designed the study, contributed to the analyses, and edited the manuscript. B.G.J.C. and Bd.G. collected the data. W.A.M.H.T. and H.L. are founders of the NEED, collected data, and helped edited the article. J.K. edited the article, takes full responsibility for the study, and acts as a guarantor. All authors have read and approved the article.

### Conflicts of interest

J.K. is a major shareholder, director, and chief medical officer of Tapa Healthcare DAC. The other authors have no potential conflicts of interest.

## Supplementary Material


