# Supplementary material for: The prediction of 24-h mortality by the respiratory rate and oxygenation index compared with National Early Warning Score in emergency department patients: an observational study
Source: Eur J Emerg Med. 2022 Dec 5;30(2):110–6. doi: 10.1097/MEJ.0000000000000989 (PMC9946171; doi:10.1097/MEJ.0000000000000989)
Supplement: Supplementary file 1 [file ejem-30-110-s001.pdf]

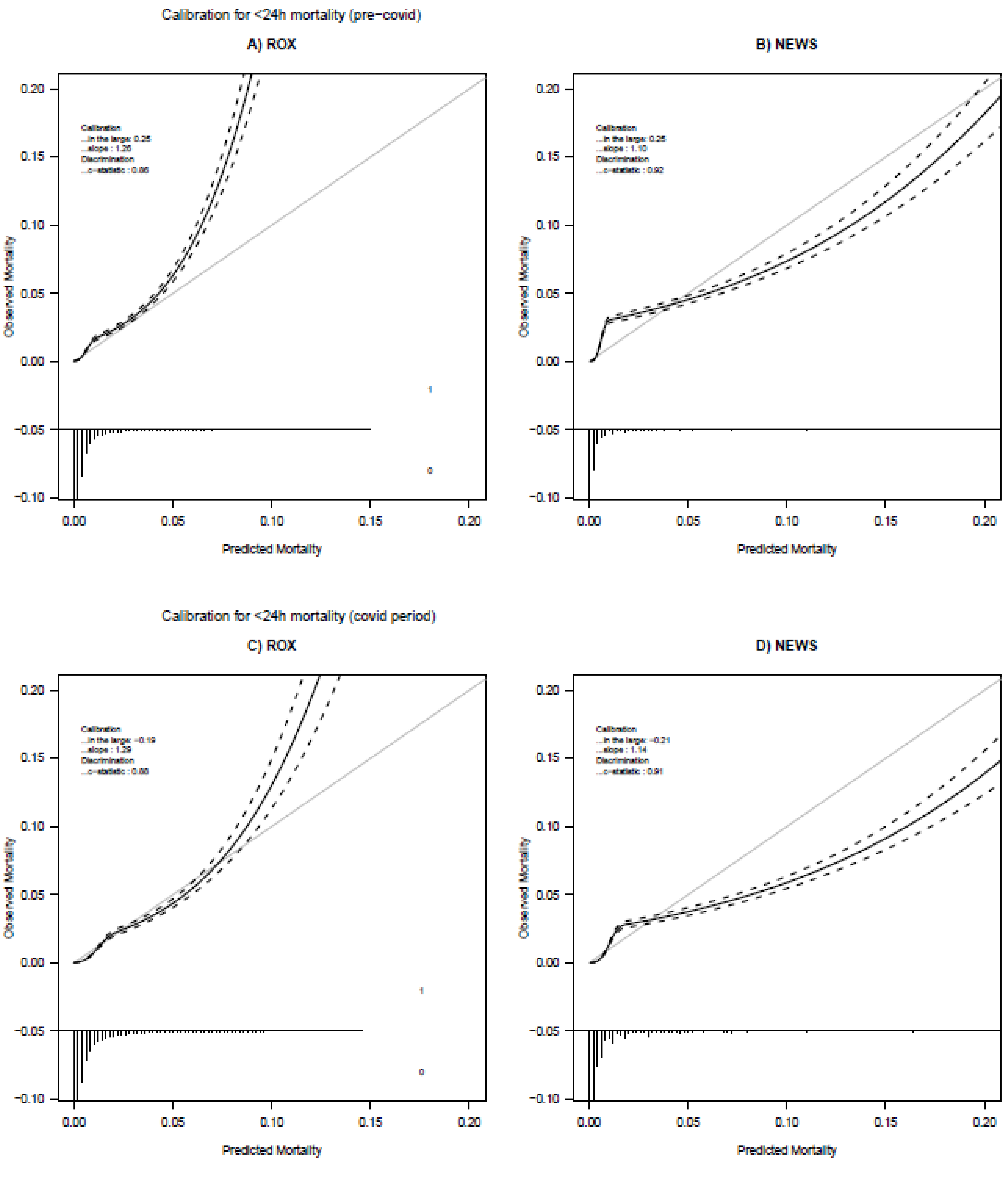

Calibration for <24h mortality (pre−covid)
A) ROX
Calibration
...in the large: 0.25
...slope : 1.26
Discrimination
B) NEWS
Calibration
...in the large: 0.25
...slope : 1.10
Discrimination
...c−statistic : 0.92
Calibration for <24h mortality (covid period)
C) ROX
Calibration
...in the large: −0.19
...slope : 1.29
Discrimination
D) NEWS
Calibration
...in the large: −0.21
...slope : 1.14
Discrimination
...c−statistic : 0.91
Observed Mortality
Predicted Mortality
0.00
0.05
0.10
0.15
0.20
−0.05
−0.10
1
0

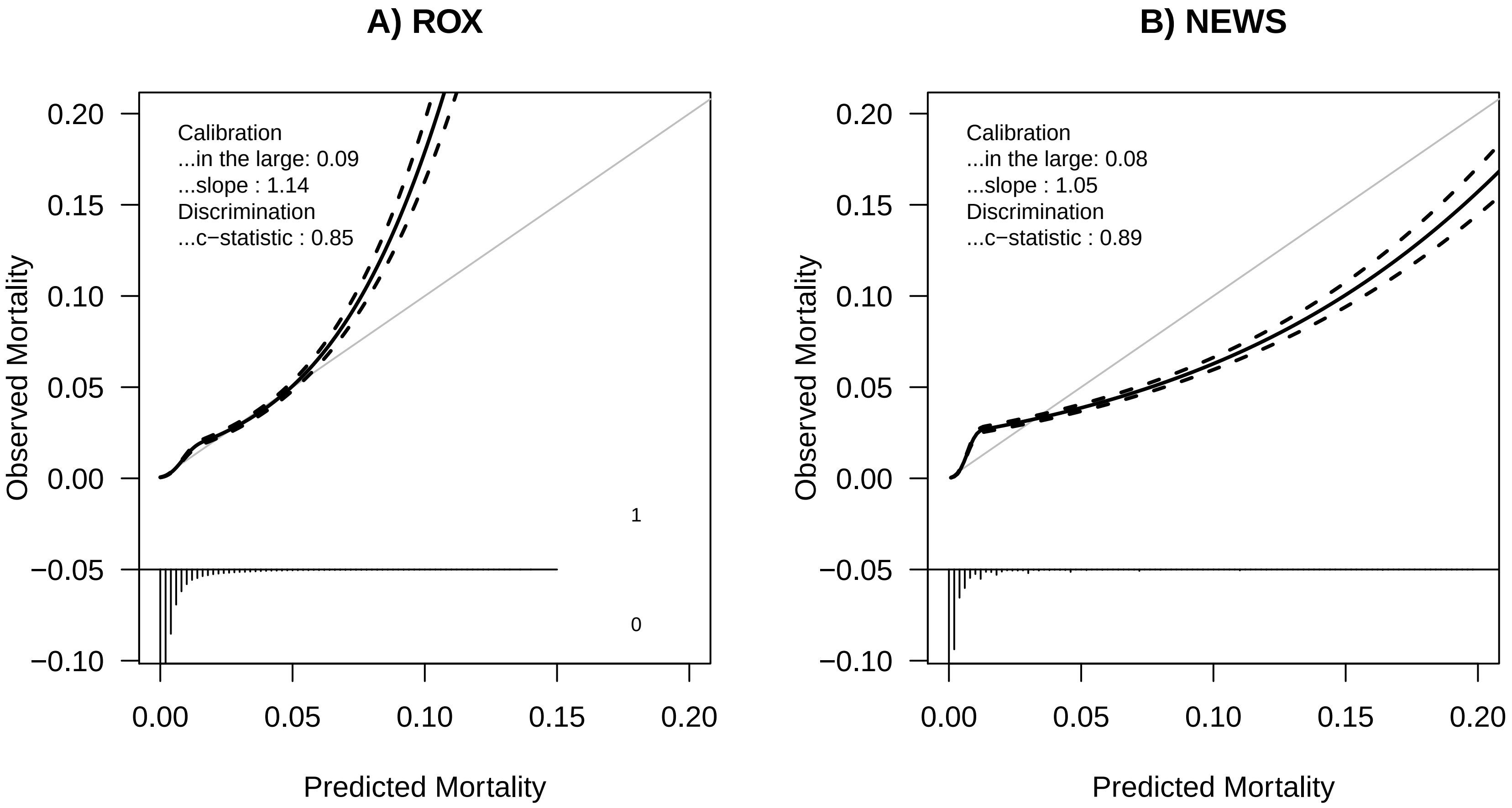
A) ROX
B) NEWS
Calibration
...in the large: 0.09
...slope : 1.14
Discrimination
...c−statistic : 0.85
Calibration
...in the large: 0.08
...slope : 1.05
Discrimination
...c−statistic : 0.89
Observed Mortality
Predicted Mortality
1
0

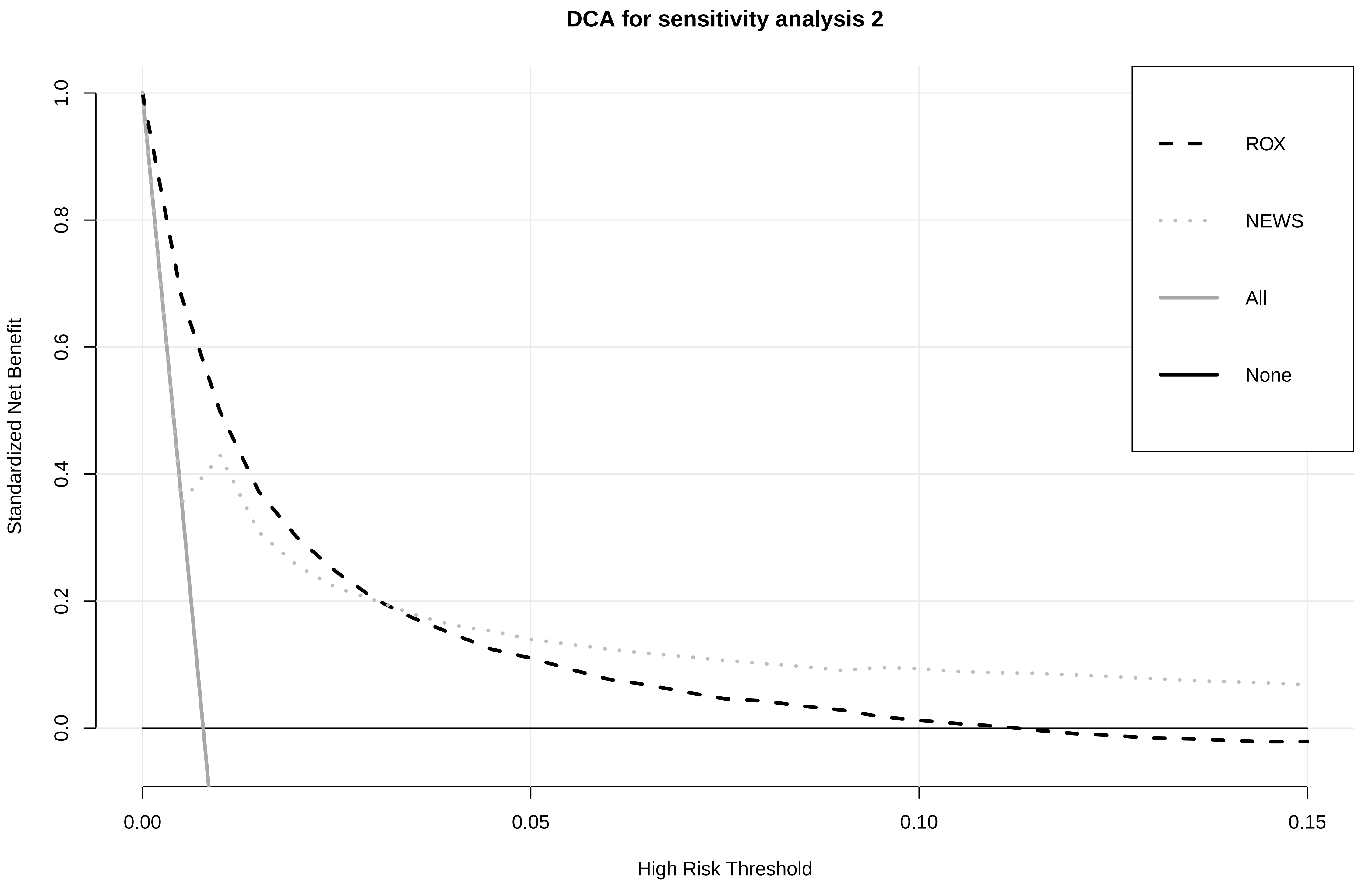
DCA for sensitivity analysis 2
Standardized Net Benefit
1.0
0.8
0.6
0.4
0.2
0.0
0.00
0.05
0.10
0.15
High Risk Threshold
ROX
NEWS
All
None
